# Role of whole-body MRI for treatment response assessment in multiple myeloma: comparison between clinical response and imaging response

**DOI:** 10.1186/s40644-020-0293-6

**Published:** 2020-01-30

**Authors:** Ho Young Park, Kyung Won Kim, Min A. Yoon, Min Hee Lee, Eun Jin Chae, Jeong Hyun Lee, Hye Won Chung, Dok Hyun Yoon

**Affiliations:** 1 Department of Radiology and Research Institute of Radiology, Asan Image Metrics, Clinical Trial Center, Asan Medical Center, University of Ulsan College of Medicine, 88 Olympic-ro 43-gil, Songpa-gu, Seoul, 05505 South Korea; 2 Department of Oncology, Asan Medical Center, University of Ulsan College of Medicine, Seoul, South Korea

**Keywords:** Multiple myeloma, Whole-body imaging, Magnetic resonance imaging, Response assessment

## Abstract

**Background:**

Whole-body MRI (WB-MRI) including diffusion-weighted image (DWI) have been widely used in patients with multiple myeloma. However, evidence for the value of WB-MRI in the evaluation of treatment response remains sparse. Therefore, we evaluated the role of WB-MRI in the response assessment.

**Methods:**

In our WB-MRI registry, we searched multiple myeloma patients treated with chemotherapy who underwent both baseline and follow-up WB-MRI scans. Clinical responses were categorized as complete response (CR), partial response (PR), stable disease (SD), or progressive disease (PD), using IMWG criteria. Using RECIST 1.1, MD Anderson (MDA) criteria, and MDA-DWI criteria, imaging responses on WB-MRI were rated as CR, PR, SD, or PD by two radiologists independently. Then, discrepancy cases were resolved by consensus. Weighted Kappa analysis was performed to evaluate agreement between the imaging and clinical responses. The diagnostic accuracy of image responses in the evaluation of clinical CR, objective response (CR and PR), and PD was calculated.

**Results:**

Forty-two eligible patients were included. There was moderate agreement between imaging and clinical responses (κ = 0.54 for RECIST 1.1, κ = 0.58 for MDA criteria, κ = 0.69 for MDA-DWI criteria). WB-MRI showed excellent diagnostic accuracy in assessment of clinical PD (sensitivity 88.9%, specificity 94.7%, positive predictive value [PPV] 84.2%, negative predictive value [NPV] 96.4% in all three imaging criteria). By contrast, WB-MRI showed low accuracy in assessment of clinical CR (sensitivity 4.5%, specificity 98.1%, PPV 50.0%, NPV 71.2% in all three imaging criteria). As to the clinical objective response, the diagnostic accuracy was higher in MDA-DWI criteria than RECIST 1.1 and MDA criteria (sensitivity/specificity/PPV/NPV, 84.2%/94.4%/98.0%/65.4, 54.4%/100%/100%/40.9, and 61.4%/94.4%/97.2%/43.6%, respectively).

**Conclusions:**

In the imaging response assessment of multiple myeloma, WB-MRI showed excellent performance in the evaluation of PD, but not in the assessment of CR or objective response. When adding DWI to imaging response criteria, diagnostic accuracy for objective response was improved and agreement between imaging and clinical responses was increased.

## Background

Recent advances have changed the role of imaging modalities in the diagnosis and staging of multiple myeloma (MM) [1–9]. Whole-body magnetic resonance imaging (WB-MRI) is of particular note because of its excellent soft tissue contrast, which allows evaluation of the bone marrow space, and has improved the prognosis of MM by facilitating the early detection of bone marrow lesions [10–16]. However, the use of WB-MRI as a response assessment tool is still the subject of debate. Despite its high diagnostic performance, WB-MRI is subject to the limitation that it may show persistent non-viable lesions after treatment [5, 17, 18].

In 2015, the International Myeloma Working Group (IMWG) made a consensus statement on the role of WB-MRI for MM [5]. In terms of response assessment, it states “MRI might help in the better definition of CR,” and also “the systemic performance of MRI for the follow-up of patients in the absence of clinical indications is not recommended” [5]. However, the consensus statement itself was evidence level D (panel consensus). Indeed, there have been only a few small studies evaluating the diagnostic performance of WB-MRI in response assessment [19–23], and, surprisingly, no study has evaluated the role of WB-MRI in differentiating progressive disease (PD) from non-PD, which is one of the most important issues in clinical oncology.

The recently updated NCCN guideline version 3.2019 also used the rather vague term “when clinically indicated” to describe the indication for WB-MRI during follow-up after primary therapy [24]. The phrase “clinically indicated” might mean PD on clinical assessment. However, evidence for the exact relationship between radiological response and clinical response is also sparse, indicating the need for further study to clarify the role of WB-MRI.

Furthermore, in respect to multiple myeloma, there remains another critical issue with imaging criteria that has not yet been fully addressed. In terms of anatomic imaging response criteria, RECIST 1.1 is currently regarded as the standard assessment tool [25]. However, RECIST 1.1 only focuses on solid tumor, which raises an issue as to whether it is relevant or not for use in MM, which is a hematologic malignancy that mainly presents with lytic lesions in bone marrow [11, 26, 27]. Thus, the MD Anderson (MDA) criteria have been proposed for response assessment in bone metastases, including in multiple myeloma [28]. The role of RECIST 1.1 and MDA in the assessment of treatment response in multiple myeloma has not been evaluated yet. Regarding functional imaging, recent studies have proposed the role diffusion-weighted image in response evaluation and suggested its use in clinical practice [2, 16, 17, 29]. Several studies have shown that increase in ADC value during treatment is correlated with good response [21, 29, 30]. However, no study has elucidated the added benefit of DWI by comparing anatomic imaging response criteria with DWI and without DWI.

To this end, we performed this study to evaluate the agreements and discrepancies between imaging response and clinical response in MM. In addition, we aimed to evaluate the diagnostic performance of WB-MRI for assessing treatment response, with the clinical response being set as the reference standard. For the imaging response assessment, we used the Response Evaluation Criteria in Solid Tumors (RECIST) 1.1, MD Anderson (MDA) criteria and MDA with DWI criteria.

## Methods

This retrospective study was approved by our institutional review board, and the requirement for written informed consent was waived. The STARD 2015 guideline for reporting diagnostic performance was followed.

### Patients

Our institution started to perform WB-MRI for multiple myeloma patients in March 2015 and established a WB-MRI registry. This registry was searched for eligible patients according to the inclusion and exclusion criteria. The inclusion criteria were as follows: [1] patients who were diagnosed with multiple myeloma between March 2015 and March 2018 at our institution [2]; patients who underwent systemic chemotherapy with or without autologous stem cell transplantation (ASCT); and [3] patients who underwent at least two consecutive WB-MRI, one at baseline and one during follow-up. The exclusion criteria included: [1] patients with no evaluable disease according to the IMWG criteria, and [2] the presence of other known malignancy.

### Image acquisition

The WB-MRI scans evaluated in this study were performed on the same 3-T unit (Skyra, Siemens Healthineers), using a 16-channel head and neck coil, a 64-channel body coil, and four body surface coils. A multiple-station acquisition was implemented to cover the full body (vertex to feet). The full scanning sequences and their parameters are summarized in Table 1.

**Table 1:** WB-MRI sequence parameters

	T2 Coronal	T1 Coronal	T2 Sagittal	T1 Sagittal	DWI Coronal	T1 Coronal	T1 Axial	T1 Sagittal	T1 Axial brain
Contrast^b^	No	No	No	No	No	Yes	Yes	Yes	Yes
Anatomic coverage	Whole body^a^	Whole body	Whole spine (C, T, L spine)	Whole spine (C, T, L spine)	Torso^c^	Whole body^a^	Torso^c^	Whole spine (C, T, L spine)	Brain
Sequences	2D, FS, TSE	3D, VIBE	2D, TSE	2D, TSE	2D EPI	3D VIBE	3D VIBE	2D, TSE	2D spin-echo
TE (ms)	91	1.4	77	10	63	1.3	1.3	10	9.6
TR (ms)	5260	3.1	3610	520	4500	4.4	4.0	520	500
Flip angle (°)	130	9.0	150	120	90	13.0	9.0	120	70
Echo train length	17	1	17	4	41	1	2	4	1
Number of slices	55	55	20	20	100	55	280	20	20
Slice thickness (mm)	5	5	3.9	3.9	2.5	5	3	3.9	5

^a^Whole-body ranges from cranial vertex to feet

^b^Contrast enhancement is performed under clinicians’ approval only when the renal function is within normal range

^c^Torso includes neck, chest, abdomen, and pelvis

Abbreviations: DWI = diffusion weighted imaging; FS = fat suppression; TSE = turbo spin echo; VIBE = volumetric interpolated breath-hold examination; EPI = echo-planar image

The unenhanced MR imaging sequences consisted of coronal T2 turbo spin-echo (TSE) with fat saturation, coronal T1 volumetric interpolated breath-hold examination (VIBE), sagittal T2 TSE, sagittal T1 TSE, and diffusion-weighted echo-planar imaging with two different b-values (b50, b900). After contrast injection, axial T1 VIBE Dixon (neck–chest, abdomen, pelvis), coronal T1 VIBE Dixon, T1 TSE sagittal, and T1 spin-echo (SE) axial sequences were obtained.

### Medical information review

The patients’ medical information was reviewed from their medical records, including treatment history, clinical stability, and laboratory test results. Medical information for the evaluation of clinical response was recorded in all patients at each MR visit. The time interval between recording of the medical information and the WB-MRI scanning was less than 2 weeks in all cases. The laboratory test results included plasma cell percentage on bone marrow biopsy, serum and urine M-protein, serum-free light chain (FLC) ratio, absolute FLC difference, beta-2 microglobulin, albumin, calcium, creatinine, and hemoglobin level.

### Clinical response assessment

A clinical researcher (K.L., general physician with 1 year of experience) evaluated the clinical responses of the patients at each MR visit using the IMWG criteria. The IMWG criteria are based on changes in M-protein as a major indicator for response assessment. The response categories include complete response (CR), very good partial response (VGPR), partial response (PR), minimal response (MR), stable disease (SD), and progressive disease (PD) [1]. Detailed explanations of each response category are provided in Additional file 1: Table S1. To analyze the associations between clinical and imaging responses, the clinical responses were simplified to seroCR, seroPR (VGPR+PR + MR), seroSD, and seroPD, where the prefix ‘sero’ indicates IMWG criteria based solely on laboratory data, excluding any imaging components.

### Imaging response assessment

Five MR imaging patterns of marrow involvement have been described in multiple myeloma [3]: [1] normal appearance of the bone marrow despite minor microscopic plasma cell infiltration, [2] focal involvement, [3] homogeneous diffuse infiltration, [4] combined diffuse and focal infiltration, and [5] ‘salt and pepper’ pattern with inhomogeneous bone marrow with interposition of fat islands.

Two radiologists (K.W.K., 13 years of experience; H.Y.P., 4 years of experience) independently evaluated the imaging response at each MR visit using the RECIST 1.1, MDA criteria, and MDA-DWI criteria, without knowledge of the clinical response. A web-based electronic case report form (eCRF) was used for the independent blind reviews. If a discrepancy was detected between the two radiologists, a consensus was made through image review. The discrepancy rate was calculated and the reasons for the discrepancy were recorded in the eCRF form.

While performing the imaging response assessment, focal involvement of bone marrow was considered as the target lesion in most cases because it is easy to measure the size of such lesions. Other bone marrow involvement patterns such as diffuse or ‘salt and pepper’ patterns were regarded as non-target lesions.

#### RECIST 1.1

According to RECIST 1.1, a target lesion for soft tissue is a well-defined lesion ≥10 mm in the longest axis, while for lymph node it is ≥15 mm in the shortest axis [25]. The largest sum of diameters (SoD) of five target lesions is evaluated, with a maximum of two lesions per organ [25]. RECIST 1.1 contains the four response categories of CR, PR, PD, and SD. The detailed response evaluations are summarized in Additional file 1: Table S2. In this study, the two maximal focal bone marrow lesions exceeding 10 mm in size were counted as target lesions. Extramedullary soft tissue myelomas, if present, were measured in an identical manner. Non-target lesions were also evaluated at every MR visit.

#### MD Anderson criteria

The MDA criteria are bone-specific response criteria developed by The University of Texas MD Anderson Cancer Center in 2004 for evaluation of bone metastases [28, 31]. They include objective measurement of the perpendicular diameters of measurable lesions and subjective evaluation of unmeasurable lesions [28]. The MDA criteria divides responses into the four categories of CR, PR, PD, and SD [28]. We adjusted the MDA criteria for response evaluation in multiple myeloma, choosing the two maximal target lesions in the bone marrow, and regarded all remaining bone marrow lesions as non-target lesions. If there were discrepancies between the responses of target and non-target lesions, the responses of the lesions that represented the bulk of the disease were followed. The detailed response criteria are described in Additional file 1: Table S3.

#### MDA-DWI criteria

To create imaging response assessment criteria based on both anatomic and functional imaging criteria, we modified MDA criteria by adding DWI criteria (MDA-DWI). Details of the DWI criteria were adopted from a recently proposed structured reporting tool for multiple myeloma, Myeloma Response Assessment and Diagnosis System (MY-RADS) [16]. Modified statements of the DWI criteria are presented in Additional file 1: Table S3. To obtain ADC value, ROI was drawn manually on high b-value image for maximal two target lesions which were selected using MDA criteria. In patient with diffuse involvement pattern, ROI was drawn in the bone region with signal alteration (i.e., vertebral body or pelvic bone). Mean ADC values of ROIs were calculated and compared between MR visits.

### Statistical analysis

The primary outcome was the agreement between the clinical response and imaging responses, which was evaluated using weighted Kappa. The secondary outcomes included the reliability of the imaging response assessment with regard to inter-reader agreement and inter-criteria agreement, evaluated using weighted Kappa.

The diagnostic accuracies of WB-MRI (sensitivity, specificity, positive predictive value, negative predictive value) in the evaluation of clinical CR, objective response (CR and PR), and PD were calculated. SPSS (version 23, IBM) and Medcalc software (version 14.8.1, Medcalc, Mariakerke, Belgium) were used for the analysis, with a significance level of *p* = 0.05 indicating statistical significance.

## Results

### Patients

**Fig. 1:**
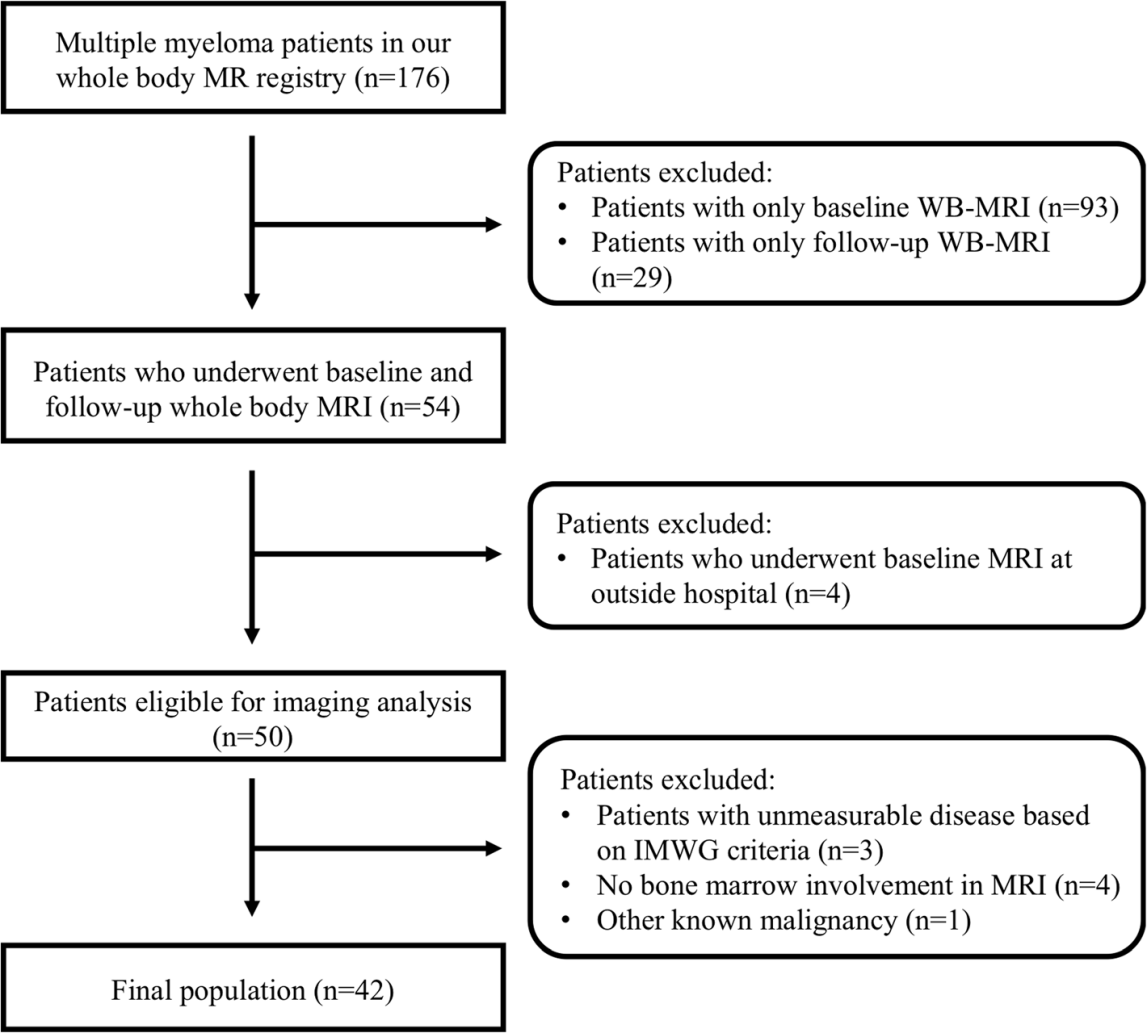
Flow chart of patient enrollment

A total of 176 patients with multiple myeloma were registered in our WB-MRI registry between March 2015 and March 2018. As summarized in Figs. 1, 122 patients were excluded because they underwent only a single MR examination, either at baseline or during follow-up. We also excluded four patients who underwent baseline WB-MRI at outside hospitals, and another eight patients with either no evaluable disease according to IWMG criteria or WB-MRI, or the presence of other known malignancies. Finally, 42 consecutive patients who met the inclusion and exclusion criteria were enrolled.

The patient demographics are shown in Table 2. The majority of the patients (*n* = 38; 90.4%) were treated with bortezomib-based systemic chemotherapy, either with VTD (bortezomib + thalidomide + dexamethasone, *n* = 28) or VMP (bortezomib + melphalan + prednisolone, *n* = 10). The rest of the patients were treated with TD (thalidomide + dexamethasone, n = 3) or DCEP (dexamethasone + cyclophosphamide + etoposide + cisplatin, n = 1). The distributions of the imaging patterns were as follows: focal (*n* = 25; 59.5%), diffuse (*n* = 5; 11.9%), focal and diffuse (n = 5; 11.9%), and salt and pepper pattern (*n* = 7; 16.7%).

**Table 2:** Patient demographics

Sex (no.)	
Male	25 (59.5%)
Female	17 (40.5%)
Median age	59.5 yrs. (38–81)
Imaging pattern on WB-MRI (no.)	
Focal	25 (59.5%)
Diffuse	5 (11.9%)
Focal and diffuse	5 (11.9%)
Salt and pepper	7 (16.7%)
Heavy chain (no.)	
IgA	4 (9.5%)
IgD	3 (7.1%)
IgG	19 (45.2%)
Light chain only	16 (38.1%)
Light chain (no.)	
Kappa	22 (52.4%)
Lambda	20 (47.6%)
International staging system (no.)	
Stage I	7 (16.7%)
Stage II	20 (47.6%)
Stage III	15 (35.7%)
Laboratory findings	
Bone marrow plasma cell (%)	39.7 ± 29.7
Serum M-protein (g/dL)	2.1 ± 2.6
Urine M-protein (mg/dL)	827.9 ± 2549.0
Serum FLC ratio^**†**^	458.9 ± 736.0
Absolute FLC difference (mg/L)	3880.7 ± 5612.6
Beta-2 microglobulin (ug/mL)	7.3 ± 8.8
Albumin (g/dL)	3.3 ± 0.7
Calcium (mg/dL)	9.3 ± 1.6
Creatinine (mg/dL)	1.7 ± 1.6
Hemoglobin (g/dL)	10.3 ± 2.1

### Agreements between imaging and clinical responses

Agreement between imaging and clinical responses was moderate when using RECIST 1.1 and MDA (κ = 0.54 and 0.58, respectively) and increased when using MDA-DWI (κ = 0.69). Cross-tabulations between the imaging and clinical responses are shown in Table 3. Notably, of the 20 cases with imaging SD on MDA criteria, 13 cases were changed to PR on MDA-DWI criteria.

**Table 3:** Imaging and clinical responses of 42 patients (75 MR visits)

		Clinical response				
		Complete response	Partial response	Stable disease	Progressive disease	Total
RECIST 1.1	Complete response	1	1	0	0	2 (2.7%)
	Partial response	11	18	0	0	29 (38.7%)
	Stable disease	9	14	0	2	25 (33.3%)
	Progressive disease	1	2	0	16	19 (25.3%)
	Total	22 (29.3%)	35 (46.7%)	0	18 (24.0%)	75
		Clinical response				
		Complete response	Partial response	Stable disease	Progressive disease	Total
MDA criteria	Complete response	1	1	0	0	2 (2.7%)
	Partial response	15	18	0	1	34 (45.3%)
	Stable disease	5	14	0	1	20 (26.7%)
	Progressive disease	1	2	0	16	19 (25.3%)
	Total	22 (29.3%)	35 (46.7%)	0	18 (24.0%)	75
		Clinical response`				
		Complete response	Partial response	Stable disease	Progressive disease	Total
MDA-DWI criteria	Complete response	1	1	0	0	2 (2.7%)
	Partial response	19	27	0	1	47 (62.7%)
	Stable disease	1	5	0	1	7 (9.3%)
	Progressive disease	1	2	0	16	19 (25.3%)
	Total	22 (29.3%)	35 (46.7%)	0	18 (24.0%)	75

In patients with seroCR and seroPR, the anatomic imaging criteria (RECIST 1.1 and MDA) showed a tendency to underestimate the response in comparison with the clinical response. Indeed, 95.4% (21 out of 22) of patients with a seroCR and 45.7% (16 out of 35) of patients with a seroPR were underestimated in the imaging response using both RECIST 1.1 and MDA criteria. When using MDA-DWI criteria, the percentage of underestimated cases was decreased in patients with seroPR (20%, 7 out of 35), while that in patients with seroCR was unchanged (95.4%, 21 out of 22).

**Fig. 2:**
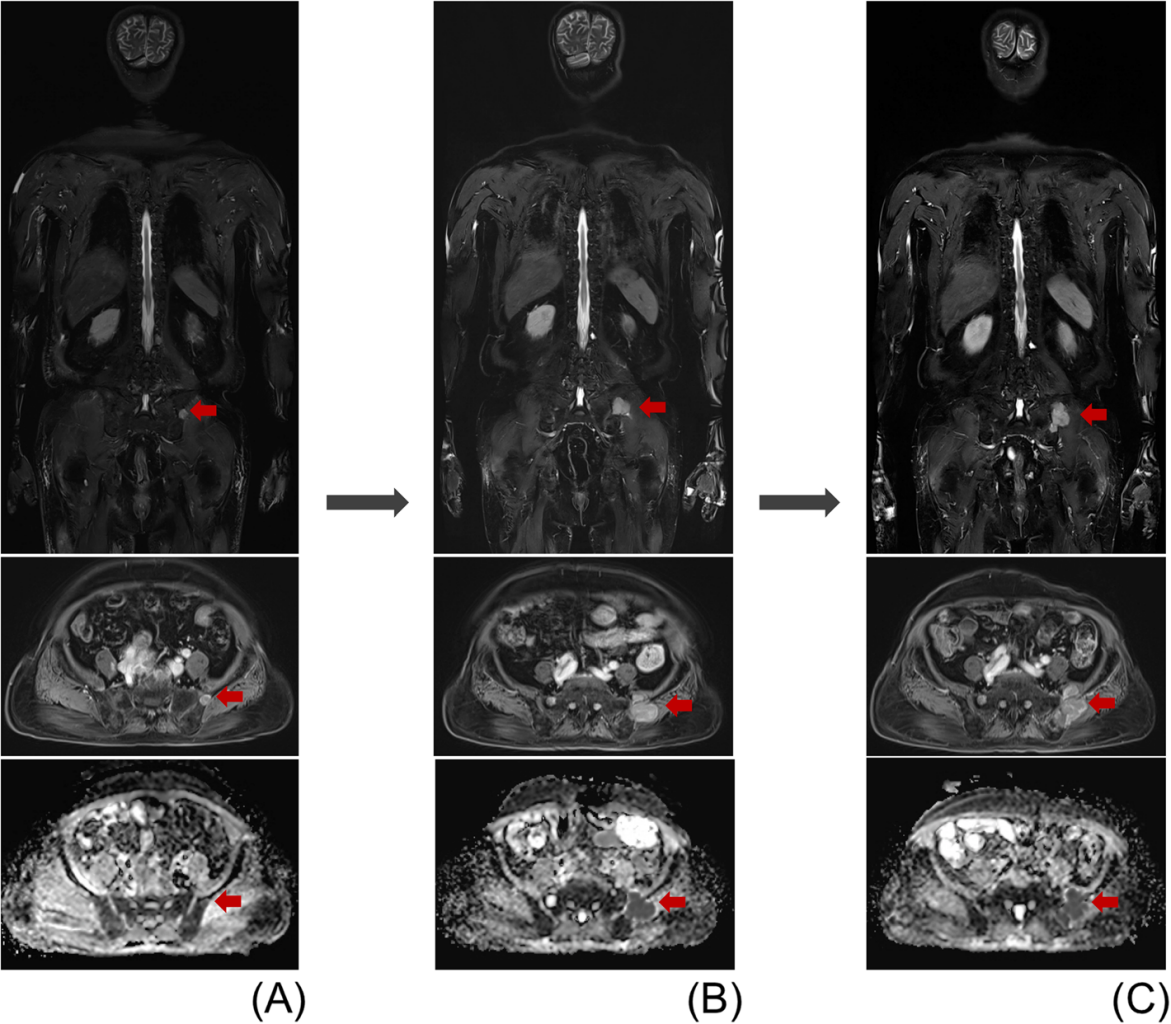
A 70-year-old male with multiple myeloma demonstrating a discrepancy between imaging response and clinical response. MRI taken before chemotherapy (**a**) shows a nodular bone marrow lesion in the left ilium with T2 hyperintensity (upper right) and contrast enhancement (middle right). It shows diffusion restriction on ADC map (mean ADC value: 0.93 × 10^− 3^ mm^2^/s) (lower right). Follow-up MRI at 3 months (**b**) and 8 months (**c**) show gradual increase in the size of the bone marrow lesion with extraosseous soft tissue extension involving the left iliacus and gluteus medius. Aggravation of diffusion restriction was noted on follow-up ADC maps (mean ADC value: 0.70 ~ 0.78 × 10^− 3^ mm^2^/s). Clinical markers improved over the same period (serum M-protein: 2.1 ➔ 0 g/dl); however, despite the improvement in the laboratory markers, the treatment regimen was changed according to the disease progression on the imaging response

In patients with seroPD, agreement with imaging PD was higher than patients with sero CR/PR. In all three criteria, four patients showed clinically significant discrepancies between clinical and imaging responses which could impact the treatment plan. A representative case is shown in Fig. 2. Brief histories of these four patients are summarized in Additional file 1: Table S4.

### Diagnostic performance of WB-MRI

**Fig. 3:**
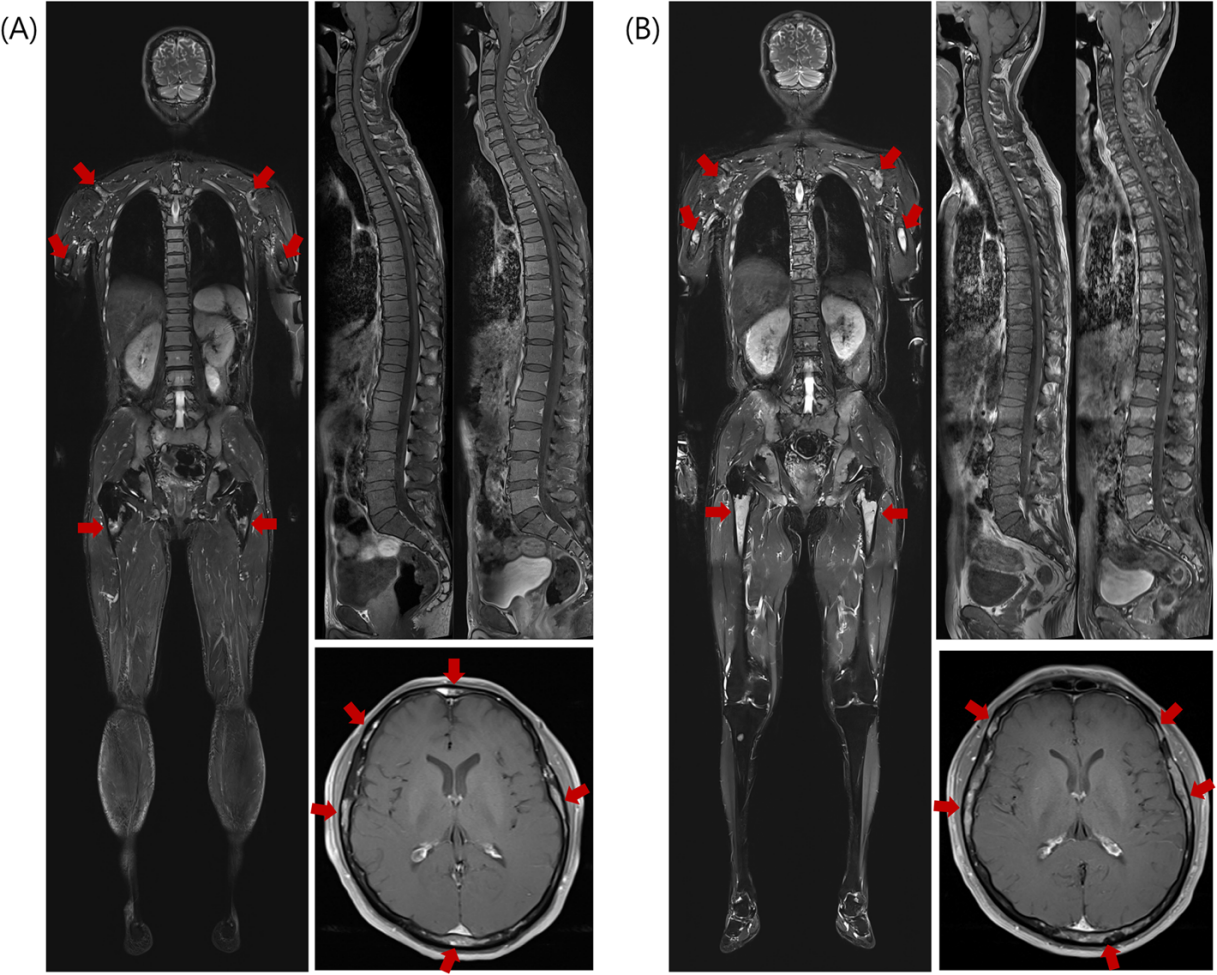
A 56-year-old male with multiple myeloma showing disease progression. Coronal T2WI (left) taken before chemotherapy (**a**) shows diffuse high signal intensity in the bone marrow (arrows). Sagittal CE T1WI (upper right) shows diffuse enhancement of bone marrow in the whole spine. Coronal enhanced T1WI (lower right) shows multifocal bone marrow enhancement in the calvarium. MRI at 14 months after initiation of chemotherapy and ASCT (**b**) shows a further increase in the signal intensity and extent of diffuse bone marrow lesions (arrows) on coronal T2WI (left) and sagittal T1WI (upper right). Axial T1WI (lower right) shows enlarged focal lesions in the calvarium, indicative of disease progression

**Fig. 4:**
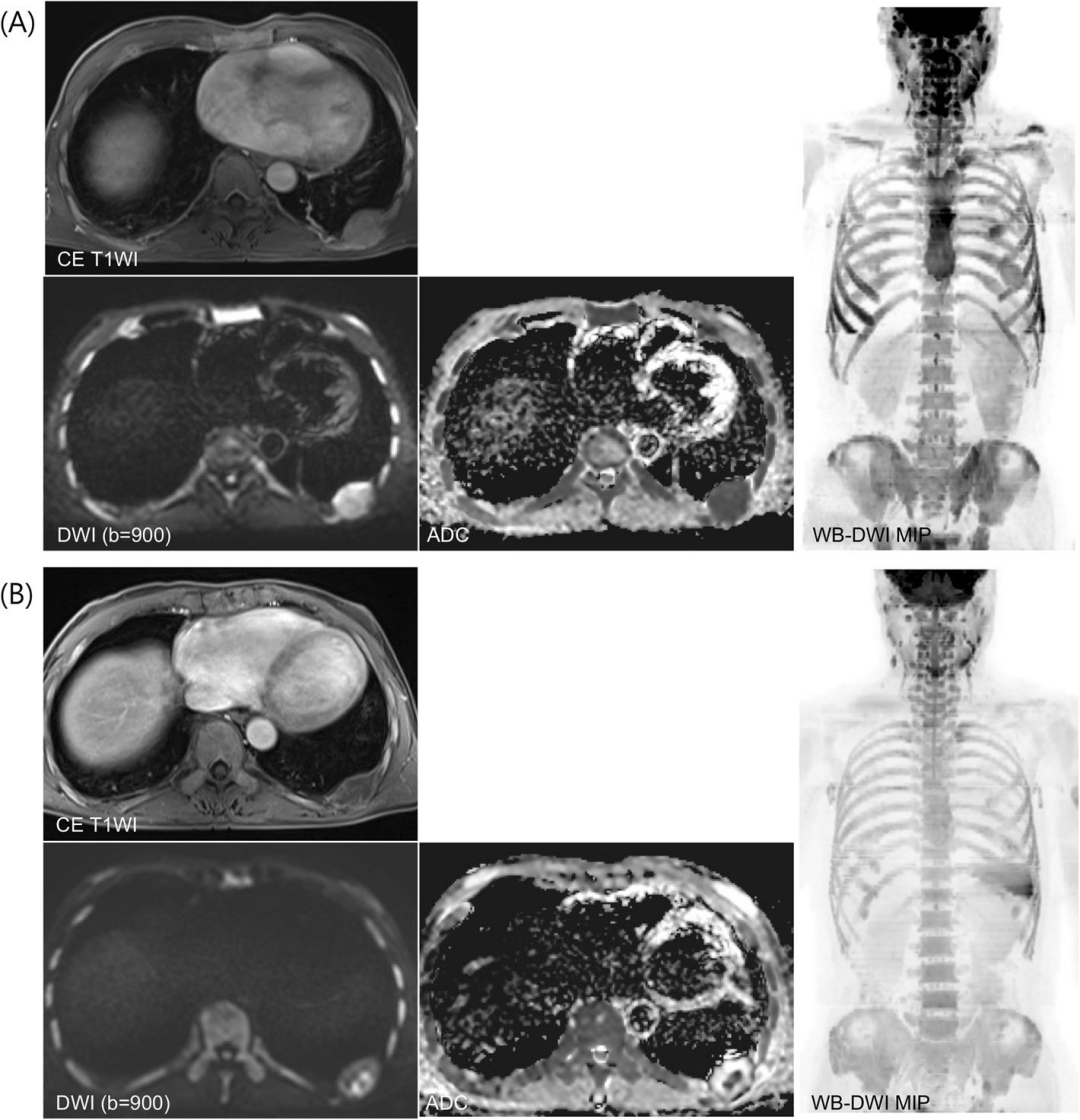
A 56-year-old male with multiple myeloma demonstrating additional benefit of DWI in the evaluation of clinical objective response. MRI taken before chemotherapy (**a**) shows a 4.0 × 3.1 cm enhancing mass at posterior arc of left 9th rib. Axial DWI (b = 900) and ADC map show marked diffusion restriction in the lesion (mean ADC value: 0.54 × 10^− 3^ mm^2^/s). Coronal diffusion MIP image (b = 900) shows diffuse high signal intensity involving whole axial skeletons, suggesting diffuse bone marrow involvement. After 4 cycles of chemotherapy, follow-up MRI (**b**) shows equivocal change in the tumor size (3.7 × 2.3 cm) in the left 9th rib. Axial DWI (b = 900) and ADC map show marked improvement of diffusion restriction in the lesion (mean ADC value: 1.54 × 10^− 3^ mm^2^/s). Coronal diffusion MIP image (b = 900) demonstrates decreased signal intensity in the axial skeletons, suggesting good response to the treatment. Based on RECIST 1.1 or MDA criteria, this patient is classified as imaging SD. However, when DWI findings are considered in MDA-DWI criteria, this patient is classified into imaging PR

Table 4 summarizes the performance of WB-MRI in the evaluation of treatment response in MM, with the clinical responses as the reference standard. WB-MRI showed high diagnostic accuracy in the assessment of clinical PD, with a sensitivity of 88.9%, specificity of 94.7%, positive predictive value (PPV) of 84.2%, and negative predictive value (NPV) of 96.4% in all three imaging criteria (Fig. 3). By contrast, WB-MRI showed low accuracy in assessment of clinical CR (sensitivity 4.5%, specificity 98.1%, PPV 50.0%, NPV 71.2% in all three imaging criteria). Regarding the clinical objective response, the diagnostic accuracy was increased in MDA-DWI criteria compared to RECIST 1.1 and MDA criteria (sensitivity 54.4%, specificity 100%, PPV 100%, NPV 40.9% for RECIST 1.1; sensitivity 61.4%, specificity 94.4%, PPV 97.2%, NPV 43.6% for MDA criteria; sensitivity 84.2%, specificity 94.4%, PPV 98.0%, NPV 65.4% for MDA-DWI criteria) (Fig. 4).

**Table 4:** Diagnostic performance of WB-MRI

	Imaging response		
	RECIST 1.1	MDA criteria	MDA-DWI criteria
Clinical CR	Sen: 4.5%, Spec: 98.1%, PPV: 50.0%, NPV: 71.2%	Sen: 4.5%, Spec: 98.1%, PPV: 50.0%, NPV: 71.2%	Sen: 4.5%, Spec: 98.1%, PPV: 50.0%, NPV: 71.2%
Clinical OR	Sen: 54.4%, Spec: 100%, PPV: 100%, NPV: 40.9%	Sen: 61.4%, Spec: 94.4%, PPV: 97.2%, NPV: 43.6%	Sen: 84.2%, Spec: 94.4%, PPV: 98.0%, NPV: 65.4%
Clinical PD	Sen: 88.9%, Spec: 94.7%, PPV: 84.2%, NPV: 96.4%	Sen: 88.9%, Spec: 94.7%, PPV: 84.2%, NPV: 96.4%	Sen: 88.9%, Spec: 94.7%, PPV: 84.2%, NPV: 96.4%

### Reliability of imaging response criteria

Analysis of inter-reader agreement showed strong agreement between the two readers in all three imaging criteria (κ = 0.82 for RECIST 1.1; κ = 0.82 for MDA criteria, κ = 0.85 for MDA-DWI criteria). The inter-reader discrepancy rates were 20.0% with RECIST 1.1, 18.7% with the MDA criteria, and 13.3% with MDA-DWI criteria. All discrepant cases were due to either different target lesion selections or discrepancy in the subjective evaluation of the tumor burden of non-target lesions.

In terms of the inter-criteria agreement, strong agreement was noted in all three imaging criteria (κ = 0.86 between the RECIST 1.1 and MDA criteria; κ = 0.85 between RECIST 1.1 and MDA-DWI criteria; κ = 0.89 between MDA criteria and MDA-DWI criteria). The discrepancy rate between RECIST 1.1 and MDA criteria was 20.0%, with the discrepancies being mainly due to non-target responses: the MDA criteria can differentiate PR and SD for non-target lesions, while RECIST 1.1 regards PR and SD as non-CR/non-PD as a whole. The discrepancy rate between MDA and MDA-DWI criteria was 17.3% due to the persistent bone marrow lesions despite decreased diffusion restriction.

## Discussion

This study investigated the relationship between imaging response and clinical response and the diagnostic accuracy of WB-MRI as a response assessment tool in MM. We demonstrated moderate agreement between clinical and anatomic imaging criteria (κ = 0.54 for RECIST 1.1, κ = 0.58 for MDA criteria), with WB-MRI having a tendency to underestimate CR or PR compared with the clinical responses. When diffusion weight image was combined, agreement between clinical and imaging response was improved (κ = 0.69 for MDA-DWI criteria). Our results are in general agreement with the 2015 IMWG consensus statement and several previous studies, in that WB-MRI may show persistent non-viable lesions in bone marrow after treatment [5, 17, 19, 32].

Compared with previous studies, this study showed WB-MRI to have low performance in the diagnosis of CR or PR. Indeed, on WB-MRI, bone marrow signal abnormalities were documented as being resolved in only one out of 22 scans that were regarded as clinical CR; we were able to detect residual lesions in bone marrow in the other 21 scans. Most of the lesions decreased in size after treatment, but a few showed no size change. In the majority of cases, the internal signal characteristics and enhancement pattern altered after treatment, with the T2 signal intensity and ADC value of the lesions being increased at follow-up and the enhancement pattern changing from solid to peripheral enhancement.

Our study showed excellent diagnostic performance of WB-MRI in the evaluation of clinical PD (sensitivity, 88.9%; specificity, 94.7% for all three imaging criteria), but poor diagnostic performance in the assessment of clinical CR (sensitivity, 4.5%; specificity, 98.1% for all three imaging criteria). This finding supports the consensus statement made by the IMWG that “routine WB-MRI is not recommended for the evaluation of treatment response”.

However, when adding DWI to anatomic WB-MRI, the diagnostic accuracy of clinical objective response (CR + PR) was compared to those of RECIST 1.1 and MDA criteria. Indeed, of the 20 cases with imaging SD on MDA criteria, 13 cases were changed to PR on MDA-DWI criteria, suggesting benefit of functional imaging when assessing persistent bone marrow lesions on anatomic imaging. These findings are in concordance with previous studies that WB-DWI can be a potential tool in treatment response evaluation of bone marrow lesions of multiple myeloma [2, 16, 33–35].

Four patients showed notable discrepancies between imaging and clinical responses. Two patients were evaluated as PD on imaging despite a clinical PR or CR. The other two patients were clinical PD, despite showing a PR or SD on imaging. Interestingly, in one patient with PD on imaging, the laboratory markers including M-protein, FLC ratio, and absolute FLC difference continued to fluctuate, while the imaging features and the patient’s bone pains worsened. The patient was managed according to the imaging response, and the chemotherapy regimen was changed. This case suggests that WB-MRI can be used to confirm PD when there is ambiguity in the clinical response or clinical suspicion of disease progression. This result is in concordance with the NCCN guideline and IMWG consensus statement that WB-MRI should be used when clinically indicated.

In our study, strong agreement was noted for inter-reader (κ = 0.82–0.85) and inter-criteria agreement (κ = 0.85–0.89). Importantly, there were no discrepancies in the evaluation of PD. Those inter-reader discrepancy cases found were due to different target lesion selections or discrepancy in the subjective evaluation of the tumor burden of non-target lesions. The discrepancy cases between RECIST 1.1 and MDA criteria were mainly due to non-target responses; the MDA criteria can differentiate PR and SD for non-target lesions, while RECIST 1.1 regards PR and SD as non-CR/non-PD as a whole. The discrepancy cases between MDA and MDA-DWI criteria were due to the persistent bone marrow lesions with variable diffusion restriction. However, as all the discrepancy cases were PR or SD, inter-reader and inter-criteria discrepancies did not impact on clinical decisions. Our study suggests that MDA-DWI criteria might be better in treatment response assessment in multiple myeloma than anatomic imaging criteria alone.

Our study has several limitations. First, this was a retrospective study, and therefore the patients showed variable intervals between MR exams. This may have impaired the strength of our results. A further prospective study design with regular MR intervals should be performed to acquire more solid data. Second, we used the RECIST 1.1, MDA criteria, and MDA-DWI criteria to evaluate imaging response, but neither of these are well-validated criteria for multiple myeloma. As the MDA criteria is bone-specific, we needed to modify the criteria to incorporate evaluation of extramedullary myeloma involvement. Further study is needed to validate th reporting system and to evaluate its clinical impact on multiple myeloma.

## Conclusion

Imaging response assessment using WB-MRI showed excellent performance in the evaluation of disease progression, but not in the assessment of complete response or objective response. When adding DWI to imaging response criteria, diagnostic accuracy for objective response was improved and agreement between imaging and clinical responses was increased.

## Supplementary information

**Additional file 1: Table S1.** International Myeloma Working Group criteria for response assessment (IMWG consensus criteria 2016). **Table S2.** RECIST 1.1 (Response Evaluation Criteria in Solid Tumor). **Table S3.** MD Anderson (MDA) criteria. **Table S4.** Cases showing a significant discrepancy between imaging response and clinical response.

## Data Availability

Not Applicable.

## Abbreviations

CR: Complete response
IMWG: International Myeloma Working Group
MDA criteria: MD Anderson criteria
MM: Multiple myeloma
NPV: Negative predictive value
PD: Progressive disease
PPV: Positive predictive value
PR: Partial response
RECIST 1.1: Response evaluation criteria in solid tumors 1.1
SD: Stable disease
WB-MRI: Whole-body magnetic resonance imaging
