# Supplementary material for: Role of whole-body MRI for treatment response assessment in multiple myeloma: comparison between clinical response and imaging response
Source: Cancer Imaging. 2020 Jan 30;20:14. doi: 10.1186/s40644-020-0293-6 (PMC6993415; doi:10.1186/s40644-020-0293-6)
Supplement: Supplementary file 1 — Additional file 1: Table S1. International Myeloma Working Group criteria for response assessment (IMWG consensus criteria 2016). Table S2. RECIST 1.1 (Response Evaluation Criteria in Solid Tumor). Table S3. MD Anderson (MDA) criteria. Table S4. Cases showing a significant discrepancy between imaging response and clinical response. [file 40644_2020_293_MOESM1_ESM.docx]

**Table S1. International Myeloma Working Group criteria for response assessment (IMWG consensus criteria 2016)**

| Complete response | - Negative immunofixation on the serum and urine and disappearance of any soft tissue plasmacytomas and <5% plasma cells in bone marrow aspirates |
| --- | --- |
| Very good partial response | - Serum and urine M-protein detectable by immunofixation but not on electrophoresis or ≥90% reduction in serum M-protein plus urine M-protein level <100 mg per 24 h |
| Partial response | - ≥50% reduction of serum M-protein plus reduction in 24 h urinary M-protein by ≥90% or to <200 mg per 24 h; - If the serum and urine M-protein are unmeasurable, a ≥50% decrease in the difference between involved and uninvolved FLC levels is required in place of the M-protein criteria; - If serum and urine M-protein are unmeasurable, and serum-free light assay is also unmeasurable, ≥50% reduction in plasma cells is required in place of M-protein, provided baseline bone marrow plasma cell percentage was ≥30%. - In addition to these criteria, if present at baseline, a ≥50% reduction in the size (SPD)* of soft tissue plasmacytomas is also required |
| Minimal response | - ≥25% but ≤49% reduction of serum M-protein and reduction in 24 h urine M-protein by 50–89%. - In addition to the above listed criteria, if present at baseline, a ≥50% reduction in the size (SPD)* of soft tissue plasmacytomas is also required |
| Stable disease | - Not recommended for use as an indicator of response; stability of disease is best described by providing the time-to-progression estimates. Not meeting criteria for complete response, very good partial response, partial response, minimal response, or progressive disease |
| Progressive disease | - Any one or more of the following criteria: - Increase of 25% from lowest confirmed response value in one or more of the following criteria:  Serum M-protein (absolute increase must be ≥0.5 g/dL);  Serum M-protein increase ≥1 g/dL, if the lowest M component was ≥5 g/dL;  Urine M-protein (absolute increase must be ≥200 mg/24 h);   In patients without measurable serum and urine M-protein levels, the difference between involved and uninvolved FLC levels (absolute increase must be >10 mg/dL);  In patients without measurable serum and urine M-protein levels and without measurable involved FLC levels, bone marrow plasma cell percentage irrespective of baseline status (absolute increase must be ≥10%);   - Appearance of a new lesion(s), ≥50% increase from nadir in SPD* of >1 lesion, or ≥50% increase in the longest diameter of a previous lesion >1 cm in short axis; - ≥50% increase in circulating plasma cells (minimum of 200 cells per μL) if this is the only measure of disease |
| *SPD: sum of products of the maximal perpendicular diameters of measured lesions. | |

**Table S2. RECIST 1.1 (Response Evaluation Criteria in Solid Tumor)**

| Minimum target lesion size: ≥ 10 mm CT + MR  ≥ 15 mm lymph node | |
| --- | --- |
| Number of measurable lesions: Up to 5 total  2 is maximum per organ | |
| **Response Criteria of Target Lesion** | |
| Complete Response (CR) | Disappearance of all target lesions. Any pathological lymph nodes (whether target or non-target) must have reduction in short axis to <10 mm. |
| Partial Response (PR) | At least a 30% decrease in the sum of diameters of target lesions, taking as reference the baseline sum diameters. |
| Progressive Disease (PD) | At least a 20% increase in the sum of diameters of target lesions, taking as reference the smallest sum on study (this includes the baseline sum if that is the smallest on study). In addition to the relative increase of 20%, the sum must also demonstrate an absolute increase of at least 5 mm. (Note: the appearance of one or more new lesions is also considered progression). |
| Stable Disease (SD) | Neither sufficient shrinkage to qualify for PR nor sufficient increase to qualify for PD, taking as reference the smallest sum diameters while on study. |
| **Response Criteria of Non-Target lesion** | |
| Complete Response (CR) | Disappearance of all non-target lesions and normalization of tumor marker level. All lymph nodes must be non-pathological in size (<10 mm short axis). |
| Non-CR/Non-PD | Persistence of one or more non-target lesion(s) and/or maintenance of tumor marker level above the normal limits. |
| Progressive Disease (PD) | Unequivocal progression of existing non-target lesions. (Note: the appearance of one or more new lesions is also considered progression). |
| Table modified from Eisenhauer EA, et al. 2009. | |

**Table S3. Modified MD Anderson (MDA) criteria and MDA-DWI criteria***

| Complete Response (CR) | - Complete sclerotic fill-in of lytic lesions on XR or CT - Normalization of bone density on XR or CT - Normalization of signal intensity on MRI - Normalization of tracer uptake on SS |
| --- | --- |
| Partial Response (PR) | - Development of a sclerotic rim or partial sclerotic fill-in of lytic lesions on XR or CT - Osteoblastic flare - Interval visualization of lesions with sclerotic rims or new sclerotic lesions in the setting of other signs of PR and absence of progressive bony disease - ≥ 50% decrease in measurable lesions on XR, CT, or MRI - ≥ 50% subjective decrease in the size of ill-defined lesions on XR, CT, or MRI - ≥ 50% subjective decrease in tracer uptake on SS - > 25% increase in ADC from baseline with corresponding decrease in normalized high b-value signal intensity; morphologic findings consistent with stable or responding decrease* |
| Progressive Disease (PD) | - 25% increase in size of measurable lesions on XR, CT, or MRI - 25% subjective increase in the size of ill-defined lesions on XR, CT, or MRI - 25% subjective increase in tracer uptake on SS - New bone metastases - No change in size but increasing signal intensity on high b-value images (with ADC values < 1400 μm^2^/sec)* - New lesions/regions of high signal intensity on high b-value images with ADC value between 600-1000 μm^2^/sec)* |
| Stable Disease (SD) | - No change - < 25% increase or < 50% decrease in the size of measurable lesions - < 25% subjective increase or < 50% subjective decrease in the size of ill-defined lesions - No new bone metastases |
| *Diffusion weighted image is incorporated into MDA criteria for MDA-DWI criteria.  Measurements are based on the sum of a perpendicular bidimensional measurement of the greatest diameters of each individual lesion. Abbreviations: XR, radiography; CT, computed tomography; SS, skeletal scintigraphy; MRI, magnetic resonance imaging. | |

**Modification of MDA criteria for our study**

|  | **MDA criteria** | **Modification** |
| --- | --- | --- |
| Target lesion | All bone lesions | Maximum of two bone marrow lesions over 1 cm |
| Extraosseous lesion | Not counted | Counted as non-target lesion |
| Overall response | If there were discrepancies between responses of measurable and non-measurable lesions, we followed the responses of the lesions that represented the bulk of the disease | If there were discrepancies between responses of target and non-target lesions, we followed the responses of the lesions that represented the bulk of the disease |

**Table S4. Cases showing a significant discrepancy between imaging response and clinical response**

| Case 1 | Imaging SD  vs  Clinical PD | A 49-year-old woman with multiple myeloma underwent four cycles of VTD (Velcade, thalidomide, dexamethasone) and ASCT. Her initial M-protein level was 3.6 g/dL. Her baseline MR showed a diffuse pattern of bone marrow involvement in the whole axial skeleton and proximal appendicular skeleton. After induction chemotherapy, her clinical response was PR and her imaging response was SD.  Following ASCT, her M-protein further decreased to a nadir of 0.4 g/dL. On the next two visits, her M-protein level rose to 1.0 g/dL, indicating a clinical response of PD. However, follow-up MR showed a minimal improvement in bone marrow involvement, indicating an imaging response of SD. |
| --- | --- | --- |
| Case 2 | Imaging SD (RECIST 1.1)/PR (MDA and MDA-DWI)  vs  Clinical PD | A 62-year-old man with multiple myeloma received four cycles of VTD (Velcade, thalidomide, dexamethasone) and ASCT. His initial M-protein level was 2.0 g/dL. Baseline MR showed multifocal bone marrow lesions in the axial skeleton. After induction chemotherapy, the clinical response was PR and imaging response was SD.  After ASCT, his M-protein level further decreased to a nadir of 0.2 g/dL. On the next two visits, his M-protein level rose to 1.1 g/dL, indicating a clinical response of PD. However, follow-up MR showed a slight decrease in the size of the bone marrow lesions, which were SD on RECIST 1.1 and PR on MDA and MDA-DWI criteria. |
| Case 3 | Imaging PD  vs  Clinical PR | A 62-year-old woman with multiple myeloma underwent four cycles of VTD (Velcade, thalidomide, dexamethasone). Her baseline MR showed a focal bone marrow lesion in the L5 vertebral body. After induction chemotherapy, her clinical response was PR, while her MR imaging response was PD. Follow-up MR showed newly appeared heterogeneous bone marrow signal intensity in the whole axial skeleton. After ASCT and thalidomide maintenance, she was diagnosed as clinical CR. |
| Case 4 | Imaging PD  vs  Clinical PR | A 70-year-old man with multiple myeloma underwent four cycles of VMP (velcade, mephalan, prednisolone). His initial M-protein level was 2.1 g/dL. Baseline MR showed multifocal bone marrow lesions in the axial and appendicular skeleton. After four cycles of chemotherapy, the M-protein level dropped to 0.2 g/dL, indicating clinical PR. However, his 2nd and 3rd follow-up MR showed an increased extent of bone marrow lesions, suggesting imaging PD. During the follow-up period, the M-protein level further decreased to undetectable. On the basis of the MR findings, the oncologist changed the chemotherapy regimen to Rd (Renalidomide, dexamethasone). |
